# The Effect of Oxygen and Micronutrient Composition of Cell Growth Media on Cancer Cell Bioenergetics and Mitochondrial Networks

**DOI:** 10.3390/biom11081177

**Published:** 2021-08-09

**Authors:** Fereshteh Moradi, Christopher Moffatt, Jeffrey A. Stuart

**Affiliations:** Department of Biological Sciences, Brock University, St. Catharines, ON L2S 3A12, Canada; cmoffatt2@brocku.ca

**Keywords:** metabolism, glycolysis, mitochondria, cell culture, mitochondrial networks, oxidative phosphorylation

## Abstract

Cancer cell culture is routinely performed under superphysiologic O_2_ levels and in media such as Dulbecco’s Modified Eagle Medium (DMEM) with nutrient composition dissimilar to mammalian extracellular fluid. Recently developed cell culture media (e.g., Plasmax, Human Plasma-Like Medium (HPLM)), which are modeled on the metabolite composition of human blood plasma, have been shown to shift key cellular activities in several cancer cell lines. Similar effects have been reported with respect to O_2_ levels in cell culture. Given these observations, we investigated how media composition and O_2_ levels affect cellular energy metabolism and mitochondria network structure in MCF7, SaOS2, LNCaP, and Huh7 cells. Cells were cultured in physiologic (5%) or standard (18%) O_2_ levels, and in physiologic (Plasmax) or standard cell culture media (DMEM). We show that both O_2_ levels and media composition significantly affect mitochondrial abundance and network structure, concomitantly with changes in cellular bioenergetics. Extracellular acidification rate (ECAR), a proxy for glycolytic activity, was generally higher in cells cultured in DMEM while oxygen consumption rates (OCR) were lower. This effect of media on energy metabolism is an important consideration for the study of cancer drugs that target aspects of energy metabolism, including lactate dehydrogenase activity.

## 1. Introduction

Standard cell culture procedures originating in the mid-20th century remain widely used today for investigating cell biology, including toxicity testing, and drug development. However, it has become increasingly clear that media composition, including oxygen, affects many aspects of cell biology, including metabolism and drug efficacy [1,2,3]. Cancer drugs are typically discovered and studied, at least initially, using cell culture models, and the media environment has been shown to affect the outcome of these studies (e.g., [3,4]). The most commonly used medium for culturing human cancer cells is Dulbecco’s Modified Eagle Medium (DMEM) [5]. DMEM has a base nutrient formulation substantially different from human plasma [6], but can also be formulated with a range of glucose, pyruvate, and L-glutamine concentrations. Specific formulations used are often not reported. The recent formulation of more “physiologic” media, such as Human Plasma-Like Medium (HPLM) [3] and Plasmax [4], which are based on the human plasma metabolome [7], has focused attention on this topic.

Similarly, under standard cell culture conditions, O_2_ is unregulated and equilibrates in the CO_2_ incubator at 18–19%. This is much higher than the 1–6% O_2_ levels that have been measured in mammalian tissues in vivo (see [8] for a comprehensive review of this topic). Several regulatory systems within human cells directly sense oxygen tensions and consequently influence physiology. Despite that, almost all present knowledge (including the initial characterizations of HPLM and Plasmax) related to the action of various stimuli (e.g., cytokines, growth factors, drugs) on cancer cells (e.g., [9,10]) is based on experiments done under non-physiological, near-atmospheric oxygen levels.

Media nutrients and O_2_ in culture have broad effects on cancer cell metabolism and these influence experimental outcomes [3,4,11,12,13]. For example, culture in HPLM significantly changes the cellular redox state relative to culture of the same cells in RPMI [3]. Physiological levels of uric acid in HPLM influence pyrimidine synthesis and remarkably reduce the efficacy of 5-fluorouracil (common chemotherapeutic drug) [3]. Consistent with that, we have shown that the concentration of even one single constituent (e.g., glucose concentration in DMEM) in culture medium significantly affected the effects of two well-characterized anti-cancer molecules: resveratrol and rapamycin on multiple human cancer cell lines (e.g., LNCaP, Huh7) [5].

Interestingly, in triple negative breast cancer cells (TNBC), pyruvate triggers a pseudohypoxic response even under atmospheric oxygen, leading to stabilization of hypoxia-inducible factor 1α (HIF1α) at the concentrations found in some commercial media (0.5–1 mM). Likewise, the high concentrations of arginine in RPMI reverse the direction of the reaction catalyzed by the urea cycle enzyme, arginosuccinate lyase. In addition, the metabolic profile of TNBC spheroids grown in Plasmax for only four days resembled the metabolic landscape of orthotopic xenografts more closely than those in RPMI [4]. Collectively, these results demonstrate that physiologic media such as HPLM and Plasmax alter cellular metabolism and the response to specific drugs. However, detailed information on the effects of media on cellular bioenergetics, including mitochondrial form and function, are presently lacking.

Here, we investigated the effects of the Plasmax formulation used by VandeVoorde et al. [4] vs. DMEM, on cellular energy metabolism and mitochondrial network characteristics. Four well-studied cancer cell lines, MCF7 breast cancer cells, SaOS2 osteosarcoma cells, LNCaP androgen-sensitive human prostate adenocarcinoma cells, and Huh7 human hepatoma cells, were included in the investigation. Cells were cultured for a minimum of two weeks to acclimatize them to one of four different cell culture conditions: (1) the most common condition used for cancer cell culture, i.e., high-glucose DMEM supplemented with 10% FBS and unregulated O_2_ (thus ~18%); (2) this same medium but with O_2_ regulated at 5%; (3) the Plasmax formulation of VandeVoorde et al. [4]; (4) this same medium but with O_2_ regulated at 5%. Under these four conditions, we evaluated cellular bioenergetics, mitochondrial abundance, and mitochondrial network morphology in all four cell lines. We found that mitochondrial form and function were significantly influenced by cell culture conditions, with both medium formulation and O_2_ levels driving different bioenergetic phenotypes. These results indicate the importance of considering culture conditions in studies of energy metabolism and mitochondrial function in vitro.

## 2. Materials and Methods

### 2.1. Materials

Dulbecco’s Modified Eagle Medium with glucose (4500 mg/L), L-glutamine, and sodium pyruvate (Cat. #D6429), and supplement-free Dulbecco’s Modified Eagle Medium powdered media (Cat. #5030), Dimethylsufoxide (DMSO), L-glutamine, HEPES ((4-(2- hydroxyethyl)-1 piperazineethane-sulfonic acid), DL-dithiothreitol (DTT), Bradford reagent, and Trypan Blue were obtained from BioShop (Burlington, ON, Canada). FBS (Cat. #F1051), nonessential amino acids (100X) (Cat. #M7145), penicillin/streptomycin solution, and 0.25% trypsin/EDTA solution were obtained from Sigma-Aldrich (St. Louis, MO, USA). Tissue culture dishes (100 × 20 mm and 60 × 15 mm) were obtained from Sarstedt, Inc (Newton, SC, USA). MatTek glass bottom dishes (35 mm) were purchased from MatTek corporation (Ashland, MA, USA). All cell lines were purchased from the American Type Culture Collection (Manassas, VA, USA). Plasmax media constituents are shown in Table S1 of [4]. α-Aminobutyrate (L-2-Aminobutyric acid; Cat. #438371), L-carnosine (Cat. #535080), and DL-3-Hydroxybutyric acid sodium salt (Cat. #A11613-06) were purchased from CEDARLANE (Burlington, ON, Canada). 2-Hydroxybutyrate (2-Hydroxybutyric acid sodium salt; Cat. #S509425) and Ammonium Metavanadate (Cat. #A634095) were purchased from Toronto Research Chemicals Inc (Toronto, ON, Canada). Agilent Seahorse Calibrant XF (Cat. #100840-000), XFe 96 Extracellular Flux assay kit (Cat. #W17619), and XFe 96 cell culture microplate (Cat. #101085-004) were purchased from Agilent Technologies (Mississauga, ON, Canada). Carbonyl cyanide-4-(trifluoromethoxy) phenylhydrazone (FCCP) (Cat. #C2920), Oligomycin (Cat. #579-13-5), Rotenone (Cat. #83-79-4), sulfite (Na_2_SO_3_ (Cat. #S0505), and Antimycin A (Cat. #1397-94-0) were purchased from Sigma-Aldrich (St. Louis, MO, USA). Unless otherwise stated, all other chemicals, reagents, and solutions (mainly used to make Plasmax) were purchased from Sigma-Aldrich (St. Louis, MO, USA), BioSHOP (Burlington, ON, Canada), or Fisher Scientific (Mississauga, ON, Canada).

### 2.2. Cell Culture

To acclimatise the cells to the experimental conditions, all cell lines were cultured in either DMEM supplemented with 10% fetal bovine serum (FBS), 2× MEM nonessential amino acid solution, or penicillin (50 I.U./mL)/streptomycin (50 μg/mL) solution, or in Plasmax media supplemented with 2.5% FBS [4] and penicillin (50 I.U./mL)/streptomycin (50 μg/mL) solution for two weeks prior to initiation of experiments (a minimum of 3–4 passages). Cells were maintained within a humidified 5% CO_2_ atmosphere at 37 °C inside one of two Forma 3110 water-jacketed incubators with O_2_ control (ThermoFisher, Waltham, MA, USA). In one incubator, O_2_ was not regulated (standard cell culture approach) and thus equilibrated to ~18% O_2_ (superphysiologic). In the other incubator, O_2_ was regulated at 5% (physiological). All media were conditioned in the corresponding incubator for 24 h prior to use to ensure equilibration with the ambient condition.

### 2.3. Sample Preparation for Measuring Cellular Respiration at 18% O_2_

Cellular respiration parameters were measured using a Seahorse Extracellular Flux Analyzer XF96 Mito Stress test (Agilent, Santa Clara, CA, USA). Cells were seeded at a concentration of 15 × 10^3^/well (MCF7, SaOS2, Huh7) and 20 × 10^3^/well (LNCaP), in a XF96-well microplate 14 h prior to OCR measurements as in [14] with some modifications. This optimal cell density per cell line was selected following titration assay to determine the optimal cell number. Cells were seeded in an 80 µL final volume of media. Wells on the four corners of the plate were medium-only to serve as background correction wells. When cells are seeded at room temperature and then transferred immediately into a tissue culture incubator (~37 °C), the environmental condition of the edge wells changes more rapidly than the environmental conditions of the interior wells with respect to temperature, humidity, and CO_2_ %. Thus, to avoid the effect of edge effect on cell growth, cells were left at room temperature for 60 min prior to transferring them to the incubator. Hydration of a flux analyser sensor cartridge probe plate was done by adding 200 µL milliQ water into each well of the utility plate and putting the cartridge back onto the utility microplate. The utility plate was then placed in a non-CO_2_ incubator at 37 °C overnight. At least one hour prior to commencing the assay, 200 μL of the Seahorse Bioscience XF96 Calibrant pH 7.4 solution was added to each well of the utility plate (after removing the milliQ water from the wells). Prior to analysis, the culture media were replaced with either unbuffered (lacking sodium bicarbonate) DMEM pH 7.4 or Plasmax pH 7.4, and cells were then allowed to equilibrate in a non-CO_2_ incubator at 37 °C to allow for precise measurement (45 min). One hour prior to measurement, the following compounds were added to the hydrated sensor cartridges: Oligomycin (1.0 μM), FCCP (1.0 μM), and rotenone/antimycin A (0.5 μM). These concentrations were selected following titration experiments (according to Agilent Seahorse XF Cell Mito Stress guideline). Then, the sensor cartridges were loaded into the Seahorse Analyzer. After calibration, the sensor cartridges were replaced with the XF96 microplates and the measurement program was resumed to obtain the following OCR parameters: basal, maximal, spare respiratory capacity, proton leak, and ATP linked. Extracellular acidification rates (ECAR) as measure of glycolysis were also measured [15]. Wave Desktop 2.6V Software (Agilent) was used for data acquisition and data analysis for assays. The OCR and ECAR values were normalised to the protein concentration per well and were presented as pmol/min/μg protein.

### 2.4. Sample Preparation for Measuring Cellular Respiration Measurements at 5% O_2_

For experiments at non-atmospheric O_2_ conditions, the XF Analyzer was placed within a gas flow controlled Hypoxic Glove chamber (Coy Laboratories, Grass Lake, MI, USA). The atmosphere was CO_2_-free and O_2_ levels were set to 5%. During the assay, temperature was controlled within the Seahorse analyser, and the air circulation within the chamber was maintained by a fan. Media and other reagents were pre-equilibrated to the 5% O_2_ atmosphere 24 h prior to the assay. To avoid reoxygenation, cell culture plates were transported from their incubators to the Hypoxic Glove chamber, where all washes were carried out. In compliance with Agilent Seahorse XF guidelines for experiments under lower-than-atmosphere O_2_ levels, seeding density was reduced to 12 × 10^3^/well (MCF7), 17 × 10^3^/well (LNCaP), 17 × 10^3^/well (SaOS2), and 12 × 10^3^/well (Huh7). Furthermore, the last column of wells (in addition to background wells) was refilled with XF calibrant solution without any cells to serve as “zero” oxygen reference, as specified by the manufacturer. To scavenge the oxygen, sodium sulfite (Na_2_SO_3_) was injected into “zero” wells and XF Hypoxia Rate Calculator Software (V2.6) was used to calculate OCR measurements. The OCR values were normalised to the protein concentration per well and were presented as pmol/min/μg protein.

### 2.5. Fluorescence Microscopy

Fluorescence micrographs of live cells were obtained using a Carl Zeiss Axio Observerl, Z1-inverted light/epifluorescence microscope equipped with ApoTome.2 optical sectioning, and a Hamamatsu ORCA-Flash 4.0 V2 digital camera (sourced through Carl Zeiss Canada, Toronto, Canada). A total of 1 × 10^3^ cells were cultured on Matek 35mm poly-D-lysine-coated glass bottom culture dishes for 48 h. Cells were switched to phenol red-free DMEM or Plasmax media containing 50 nM MitoTracker Red and maintained at 37 °C in humidified 5% CO_2_, 18% O_2_ atmosphere or 5% O_2_ for 30 min. Then, cells were washed three times with fresh medium to remove the free dye and kept in DMEM or Plasmax for imaging. Images were collected with a Plan-Apochromat 63x/NA1.40 Oil DIC M27 microscope objective. The microscope stage and objective were maintained with temperature control achieved through a TempModule S-controlled stage heater and an objective heater (PeCon, Erbach, Germany) at 37 °C, and a humidified 5% CO_2_ environment with either 18% or 5% O_2_ throughout the experiments. During experiments conducted at 5% O_2_, a humidified 5% O_2_/5% CO_2_/90% N_2_ gas mix was continuously delivered through the TempModule on-stage heater. The MitoTracker Red CMXRos signal was imaged with set excitation and emission wavelengths of 587 nm and 610 nm, respectively. The intensity of fluorescence illumination by an X-Cite 120LED light source and camera exposure times were both held constant across experiments. Z-stack series were rendered into single 2D images using the “extended depth of focus” processing tool in the Zeiss Zen 2 software. (Blue edition V2.1) Z-stacks consisted of 20 slices, each 0.25 µm apart. Maximum intensity projections were generated for each stack using the Fiji distribution of ImageJ.

### 2.6. Image Analysis

Mitochondrial morphology was analysed and quantified using the Mitochondrial Network Analysis tool (MiNA), a macro tool developed for use with the FIJI distribution of ImageJ [16]. Cells were selected randomly for each experimental condition, and fluorescence images were loaded into the program in their native format using the Bio-Formats plug-in. To improve contrast between all mitochondrial structures and background, the following pre-processing steps were performed: contrast limited adaptive histogram equalisation (CLAHE), median filtering (a 2-pixel radius), and “unsharp mask”. In the processed image, the fluorescent mitochondrial signal was subjected to thresholding in order to eliminate background signal, which could generate an artifact. A binary image was generated by thresholding, the level of which was determined using Otsu’s algorithm. From the binary, the mitochondrial footprint was calculated from the total area of mitochondrial signal-positive pixels. For the purpose of estimating the lengths of mitochondrial structures and the degree of branching, the Ridge detection plugin was applied [17,18]. Ridge detection uses florescence intensity to produce binary images, from the binary iamge morphological skeleton was generated using the Skeletonize3D plugin. The topology is then captured by the AnalyzeSkeleton plug in, the results of which are used by MiNA to generate quantitative parameters. The information extracted from the morphological skeleton is the mean of the branch lengths for each independent feature and the number of branches in each network. Mean branch length is calculated as the average length of a mitochondrial structure between two nodes. Mitochondria appear as interconnected branching networks in which branches are connected at a node. Mean network size was calculated by computing the sum of all branch lengths within an independent network and dividing this by the total number of individual networks within a cell. A total of 35 cells per condition were selected randomly from at least three separate experiments.

### 2.7. Statistical Analyses

All statistical analyses were performed using GraphPad Prism 5 software (San Diego, CA, USA). One-way ANOVAs were performed for datasets. When statistical significance in datasets was observed from one-way ANOVAs, post-hoc analysis was performed using Tukey’s honestly significant difference (HSD) test. A *p*-value of <0.05 was considered significant for all statistical tests. All data are presented as means ± standard error of mean (SEM).

## 3. Results

Here, we investigated how cell culture conditions affect cellular bioenergetics and mitochondrial form and function using four cancer cells that are routinely cultured in DMEM, or other non-physiologic commercial media, and with unregulated O_2_ that therefore equilibrates to ~18%. Both media composition and O_2_ had significant effects on these parameters, though many effects were cell line-specific.

### 3.1. Media Effects on OCR

**Figure 1:**
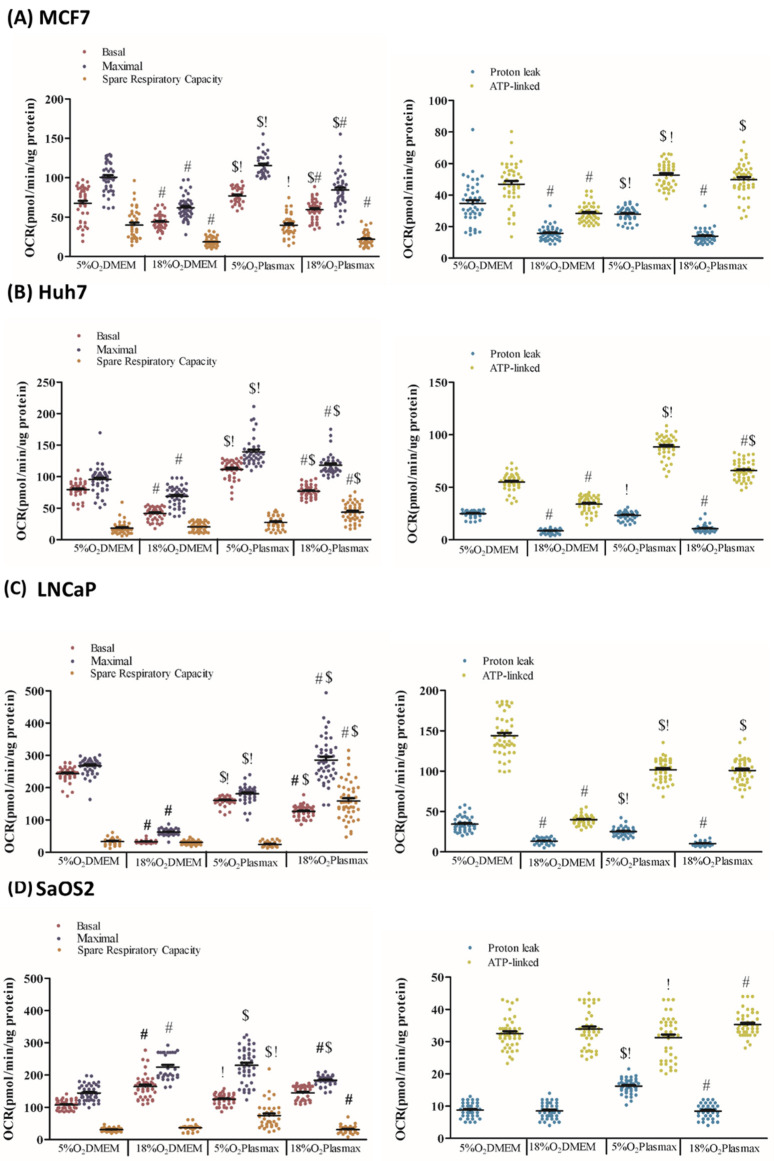
Bioenergetics profiles of cancer cell lines as a function of oxygen concentration and media composition. (**A**) MCF7, (**B**) Huh7, (**C**) LNCaP, and (**D**) SaOS2 cell lines were cultured at either 5% O_2_ or 18% O_2_ in either DMEM or Plasmax for at least two weeks prior to commencing the assay. Basal, maximal, spare respiratory capacity, proton leak, ATP-linked OCRs (normalized to protein content) were measured using a Seahorse XF96 Flux Analyzer. Data shown are means ± SEM from 3 independent experiments per condition (*n* = 43 wells per each condition for each cell line). “#” represents differences between 18% O_2_ and 5% O_2_ in the same media. “$” represents differences between Plasmax and DMEM when oxygen level is the same. “!” represents differences between 5% O_2_/Plasmax vs. 18% O_2_/DMEM. Statistical significance was determined by ANOVA followed by Tukey’s post-hoc analysis.

At 18% O_2_, MCF7 basal, maximal, and ATP-linked OCR were higher in Plasmax compared to DMEM (F_1, 176_ = 32; F_1, 176_ = 36; F_1, 176_ = 22; F_1, 176_ = 124, respectively; *p* < 0.05, Figure 1A). The same trend was observed at 5% O_2_ (F_1, 176_ = 18; F_1, 176_ = 22; F_1, 176_ = 24, respectively; *p* < 0.05, Figure 1A), though in 5% O_2_, proton leak-associated OCR was reduced in Plasmax (F_1, 176_ = 24; *p* < 0.05, Figure 1A). Broadly similar effects of media were seen in Huh7 cells. At 18% O_2_, basal (F_1, 174_ = 33), maximal (F_1, 174_ = 42), spare respiratory capacity (F_1, 174_ = 28), and ATP-linked OCR were higher in Plasmax vs. DMEM (F_1, 174_ = 19; *p* < 0.05, Figure 1B). This trend was seen also at 5% O_2_ except with no effect on spare respiratory capacity (basal (F_1, 174_ = 27); maximal (F_1, 174_ = 29); proton leak (F_1, 174_ = 41); ATP-linked (F_1, 174_ = 133); *p* < 0.05, Figure 1B).

The media effects were notably cell type-specific. While Plasmax elevated basal and maximal OCR, and spare respiratory capacity in LNCaP cells at 18% O_2_ (F_1, 176_ = 22; F_1, 176_ = 46; F_1, 176_ = 110; respectively; *p* < 0.05, Figure 1C), it had essentially the opposite effect at 5% O_2_ (basal (F_1, 176_ = 31), maximal (F_1, 176_ = 78), proton leak (F_1, 176_ = 31), and ATP-linked (F_1, 176_ = 33)). SaOS2 cells, in contrast, had a higher maximal (F_1, 175_ = 23), spare respiratory capacity (F_1, 175_ = 23), and proton leak (F_1, 175_ = 23) in Plasmax vs. DMEM (*p* < 0.05, Figure 1D) when the experiments were conducted in 5% O_2_, but these effects were largely absent at 18% O_2_ where only maximal OCR was affected, and it was reduced in Plasmax (F_1, 175_ = 23; *p* < 0.05, Figure 1D).

### 3.2. O_2_ Effects on OCR

The incubator O_2_ levels at which cells were grown and measurements performed significantly affected OCR in most of the cell lines. For example, all OCR parameters (basal (F_1, 176_ = 43), maximal (F_1, 176_ = 52), spare respiratory capacity (F_1, 176_ = 47), proton leak (F_1, 176_ = 40), ATP-linked (F_1, 176_ = 49) (*p* < 0.05, Figure 1A)) were elevated at 5% vs. 18% O_2_ in MCF7 cells grown in DMEM. Cells grown in Plasmax behaved essentially in a similar manner, with basal, maximal, and spare respirator capacity (F_1, 176_ = 42; F_1, 176_ = 55; F_1, 176_ = 72, respectively; *p* < 0.05, Figure 1A) being higher at 5% vs. 18% O_2_. Similar trends were seen with Huh7 cells. In DMEM, basal, maximal, proton leak-associated, and ATP-linked OCRs (F_1, 174_ = 49; F_1, 174_ = 42; F_1, 174_ = 50; F_1, 174_ = 72; F_1, 174_ = 68, *p* < 0.05, Figure 1B) were higher at 5% vs. 18% O_2_. Similarly, basal, maximal, proton leak, and ATP-linked OCRs (F_1, 174_ = 49; F_1, 174_ = 42; F_1, 174_ = 50; F_1, 174_ = 68, *p* < 0.05, Figure 1B) were higher at 5% vs. 18% O_2_ in Plasmax. However, spare respiratory capacity decreased at 18% vs. 5%O_2_ when Huh7 cells were grown in Plasmax (F_1, 174_ = 17, *p* < 0.05, Figure 1B).

LNCaP cells also had higher basal, maximal, proton leak, and ATP-linked OCR (F_1, 176_ = 122; F_1, 176_ = 123; F_1, 176_ = 98; F_1, 176_ = 112, respectively; *p* < 0.05, Figure 1C) at 5% vs. 18% O_2_ when grown in DMEM. However, in Plasmax only basal and proton leak-associated (F_1, 176_ = 102; F_1, 176_ = 83, respectively; *p* < 0.05, Figure 1C) were higher at 5% vs. 18% O_2_. At 18% vs. 5% O_2_, however, maximal and spare respiratory capacity (F_1, 176_ = 108; F_1, 176_ = 130, respectively; *p* < 0.05, Figure 1C) were elevated. Our data robustly indicate that LNCaP cells had greater basal and proton leak-associated OCRs at more physiologic O_2_ tension. SaOS2 cells also showed different responses to O_2_, with cells grown in DMEM having elevated basal, maximal OCRs (F_1, 175_ = 19; F_1, 175_ = 29, *p* < 0.05, Figure 1D) at 18% vs. 5% O_2_. These cells in Plasmax exhibited lower maximal, spare respiratory capacity, and proton leak-associated OCRs at 18% O_2_ (F_1, 175_ = 21; F_1, 175_ = 25; F_1, 175_ = 23, respectively; *p* < 0.05, Figure 1D).

### 3.3. OCR in Standard vs. Physiologic Cell Culture

An interesting comparison is between a standard cell culture condition (18%O_2_/DMEM) and a more physiologic cell culture (5%O_2_/Plasmax). In MCF7 cells, basal (F_1, 176_ = 64) and maximal (F_1, 176_ = 68) OCR, spare respiratory capacity (F_1, 176_ = 74), proton leak (F_1, 176_ = 60), and ATP-linked OCR (F_1, 176_ = 72; *p* < 0.05, Figure 1A) were all higher in the physiologic cell culture condition. Essentially the same results were seen in the other three cell lines. In Huh7 cells, basal, maximal, proton leak-associated, and ATP-linked OCRs (F_1, 174_ = 31; F_1, 174_ = 43; F_1, 174_ = 34; F_1, 174_ = 87, respectively; *p* < 0.05, Figure 1B) were elevated in physiologic conditions. In LNCaP cells, basal, maximal, proton leak, and ATP-linked OCRs (F_1, 176_ = 178; F_1, 176_ = 122; F_1, 176_ = 103; F_1, 176_ = 102, respectively; *p* < 0.05, Figure 1C) were elevated in physiologic cell culture. SaOS2 cells had lower basal OCR (F_1, 176_ = 22; *p* < 0.05, Figure 1D) in physiologic conditions, but spare respiratory capacity, proton leak, and ATP-linked OCR (F_1, 174_ = 31; F_1, 174_ = 18; F_1, 174_ = 20, respectively; *p* < 0.05, Figure 1D) were elevated. Overall, these comparisons suggest that physiologic conditions increase mitochondria-dependent metabolism in cancer cells.

### 3.4. O_2_ and Media Effects on ECAR

**Figure 2:**
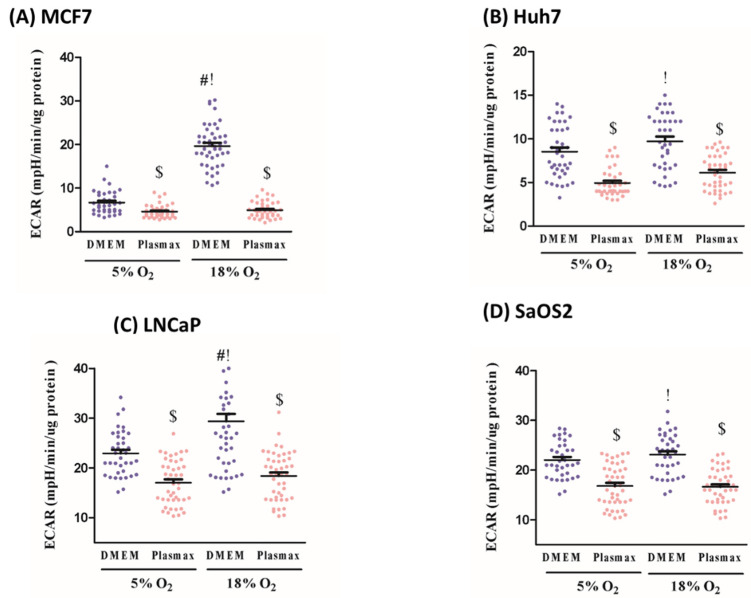
Glycolysis activity as a function of media composition and oxygen. (**A**) MCF7, (**B**) Huh7, (**C**) LNCaP, and (**D**) SaOS2 cell lines were cultured at either 5% O_2_ or 18% O_2_ in either DMEM or Plasmax for at least two weeks prior to commencing the assay. Extra cellular acidification (ECAR, normalised to protein content) was measured using a Seahorse XF96 Flux Analyzer. Data shown are means ± SEM from 3 independent experiments per condition (*n* = 43 wells per each condition for each cell line). “#” represents differences between 18%O_2_ and 5%O_2_ in the same media. “$” represents differences between Plasmax and DMEM when oxygen level is the same. “!” represents differences between 5% O_2_/Plasmax vs. 18% O_2_/DMEM. Statistical significance was determined by ANOVA followed by Tukey’s post-hoc analysis.

Measurement of the medium extracellular acidification rate (ECAR) provides an indirect analysis of the cellular glycolytic rate. All four cell lines showed higher ECAR rates in DMEM vs. Plasmax (Figure 2). In MCF7 cells at either 18% or 5% O_2_, cells in DMEM demonstrated greater glycolytic activity as shown by higher ECAR (F_1, 176_ = 117; F_1, 176_ = 16, respectively; *p* < 0.05, Figure 2A). Huh7 cells exhibited higher glycolysis in DMEM at both O_2_ levels (18% O_2_ (F_1, 174_ = 40, *p* < 0.05, Figure 2B), 5% O_2_ (F_1, 174_ = 38, *p* < 0.05, Figure 2B)). Similarly, LNCaP cells at 18% and 5% O_2_ had elevated ECAR in DMEM (F_1, 176_ = 47; F_1, 176_ = 33, respectively; *p* < 0.05, Figure 2C). SaOS2 cells also had higher ECAR in DMEM vs. Plasmax at both 18% and 5% O_2_ (F_1, 174_ = 36; F_1, 174_ = 24, respectively; *p* < 0.05, Figure 2D). Thus, in all four cancer cell lines tested here, glycolytic activity was higher in DMEM vs. Plasmax.

Finally, we investigated the effect of O_2_ on glycolysis within a given medium. MCF7 cells exhibited higher ECAR in 18% O_2_ vs. 5% O_2_ in DMEM (F_1, 176_ = 98, *p* < 0.05, Figure 2A), but there was no O_2_ effect in Plasmax. The same was seen in LNCaP cells with having higher ECAR at 18% vs. 5% O_2_ in DMEM (F_1, 176_ = 21, *p* < 0.05, Figure 2C) but not Plasmax. However, ECAR in Huh7 and SaOS2 cells was not affected by O_2_ levels (Figure 2B,D). In the comparison between standard and physiologic cell culture, MCF7, Huh7, LNCaP, and SaOS2 cell lines had elevated ECAR in the standard condition (F_1, 176_ = 129; F_1, 174_ = 79; F_1, 176_ = 62; F_1, 176_ = 59, respectively; *p* < 0.05, Figure 2A–D). Altogether, O_2_ does not seem to affect glycolysis activity when cells are in a more physiologic medium, though it was not an observation in all cell lines tested here.

### 3.5. O_2_ and Media Effects on Mitochondrial Abundance

**Figure 3:**
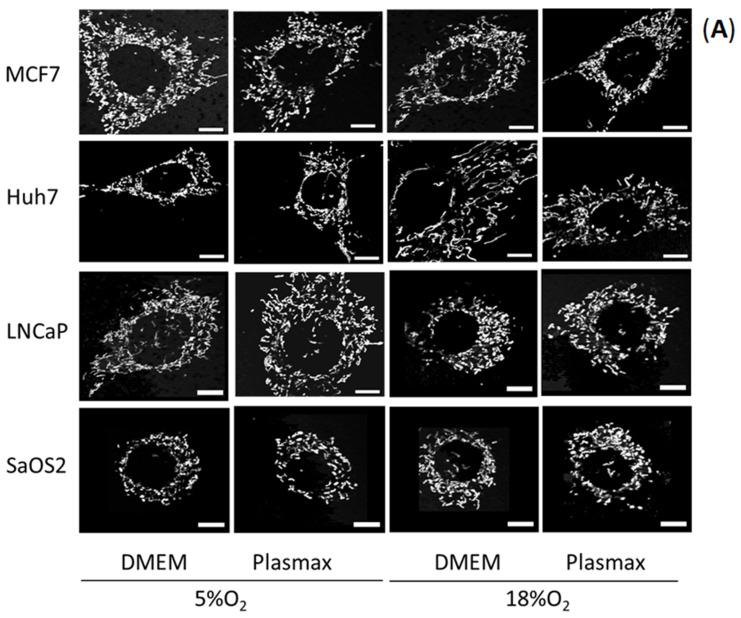

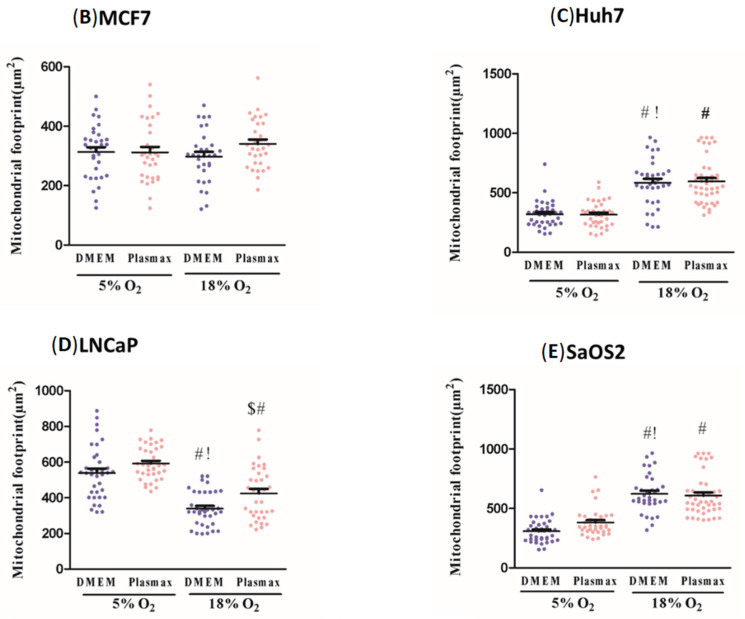
Oxygen concentration and media type influence mitochondrial abundance. (**A**) Representative images from mitochondrial network population stained by MitoTracker Red. Mitochondrial footprint (area in µm2) as a proxy of abundance in the cells was measured applying MiNA tool [16]. Feature was graphed from: (**B**) MCF7, (**C**) Huh7, (**D**) LNCaP, and (**E**) SaOS2. Cells were cultured at either 5% O_2_ or 18% O_2_ in either DMEM or Plasmax for at least two weeks prior to commencing the measurements. Data shown are means ± SEM from 3 independent experiments per condition (*n* = 35 cells per each condition for each cell line). “#” represents differences between 18% O_2_ and 5% O_2_ in the same media. “$” represents differences between Plasmax and DMEM when oxygen level is the same. “!” represents differences between 5% O_2_/Plasmax vs. 18% O_2_ /DMEM. Statistical significance was determined by ANOVA followed by Tukey’s post-hoc analysis. Scale bar size is 10 µM.

We used live-cell imaging with MitoTracker red labelling and the Mitochondrial Network Analysis Tool (MiNA) [16] to assess mitochondrial network characteristics including the “mitochondrial footprint”, which is an estimate of mitochondrial abundance in cells. At both O_2_ levels, no effect of media on mitochondrial footprint was detected in MCF7, Huh7or SaOS2 cells (Figure 3). However, a moderate higher footprint in LNCaP cells when grown in Plasmax was observed at only 5% O_2_ (F_1, 136_ = 21, *p* < 0.05, Figure 3C).

O_2_ levels had no effect on the mitochondrial footprint of MCF7 cells growing in either medium. In contrast, O_2_ had significant effects in Huh7, LNCaP, and SaOS2 cell lines (Figure 3B–D). Higher O_2_ level condition was associated with greater mitochondrial footprint in the Huh7 cell line in either cell culture medium (DMEM (F_1, 136_ = 24), Plasmax (F_1, 136_ = 26; *p* < 0.05; Figure 3B). In stark contrast, this feature in LNCaP cells was greater at 5% O_2_ in either medium (DMEM (F_1, 136_ = 20); Plasmax (F_1, 136_ = 18), *p* < 0.05, Figure 3C). SaOS2 behaved similarly to Huh7 cells where 18% O_2_ was associated with increased mitochondrial footprints in either culture medium (DMEM (F_1, 136_ = 23); Plasmax (F_1, 136_ = 17); *p* < 0.05; Figure 3D). Comparing more physiologic relevant regimen to the standard cell culture condition, Huh7 and SaOS2 cells had greater mitochondrial footprints in standard vs. physiologic (F_1, 136_ = 27; F_1, 136_ = 32, respectively; *p* < 0.05, Figure 3C,D). However, this measurement was greater in physiologic culture condition than in standard conditions in the LNCaP cell line (F_1, 136_ = 32, *p* < 0.05, Figure 3C). Overall, the O_2_ level appeared to be a more important driver of mitochondrial footprint than culture medium.

### 3.6. O_2_ and Media Effects on Mitochondrial Network Morphology

Changes in mitochondrial network morphology are associated with changes in energy metabolism. Notably, rich-nutrient environments can promote mitochondrial network fragmentation, while nutrient deprivation (lack of glucose, lipids, and amino acids) are associated with more elongated and fused mitochondrial structures [19]. We measured mitochondrial mean network size (# of branches per network) and branch mean length (in microns) to assess the extent of fusion of mitochondrial structures into longer and more highly branched networks. Once again, effects of culture condition on mitochondrial network were highly cell line-dependent.

**Figure 4:**
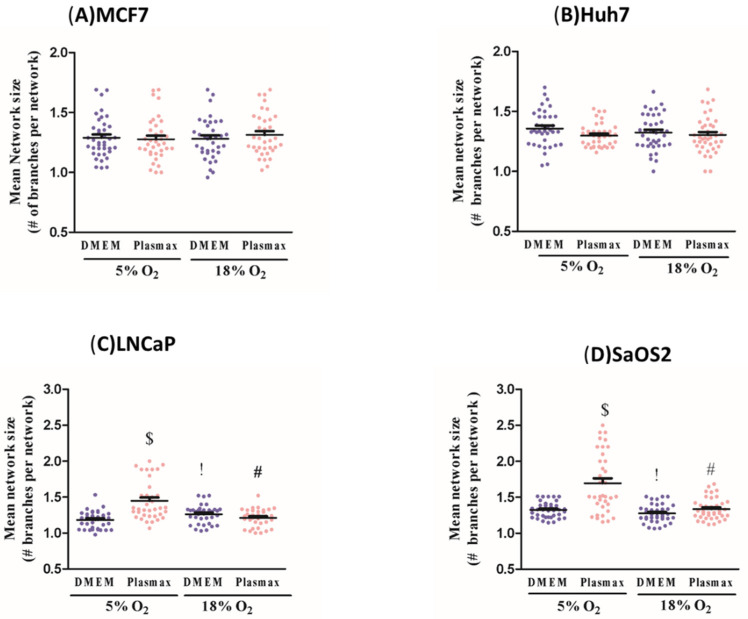
Oxygen concentration and media type influence mitochondrial network status. Mitochondrial mean network size (# of branches per network) was measured using MiNA tool in (**A**) MCF7, (**B**) Huh7 (**C**) LNCaP, and (**D**) SaOS2 cell lines. Cells were cultured at either 5% O_2_ or 18% O_2_ in either DMEM or Plasmax for at least two weeks prior to commencing the measurements. Data shown are means ± SEM from 3 independent experiments per condition (*n* = 35 cells per each condition for each cell line). “#” represents differences between 18% O_2_ and 5% O_2_ in the same media. “$” represents differences between Plasmax and DMEM when oxygen level is the same. “!” represents differences between 5% O_2_/Plasmax vs. 18% O_2_/DMEM. Statistical significance was determined by ANOVA followed by Tukey’s post-hoc analysis.

In MCF7 and Huh7 cells, neither media nor O_2_ level affected mean network size (# of branches per network) (Figure 4A,B). In LNCaP and SaOS2 cells, however, this measurement was elevated in Plasmax vs. DMEM at 5%O_2_ with no significant difference at 18% O_2_ (F_1, 136_ = 31; F_1, 136_ = 28, respectively; *p* < 0.05, Figure 4C,D). A more physiologic O_2_ level (5%) was associated with greater mean network size in only the Plasmax regimen ((LNCaP) F_1, 136_ = 28; (SaOS2) F_1, 136_ = 24, *p* < 0.05, Figure 4C,D). Mean network size was also higher in more physiologic condition than in common cell culture in both LNCaP and SaOS2 cell lines (F_1, 136_ = 23; F_1, 136_ = 19, respectively; *p* < 0.05; Figure 4C,D). Overall, mitochondria seem to be affected by cell culture condition in a cell type-specific manner with a greater fused mitochondrial network in cells grown in Plasmax and 5% O_2_.

**Figure 5:**
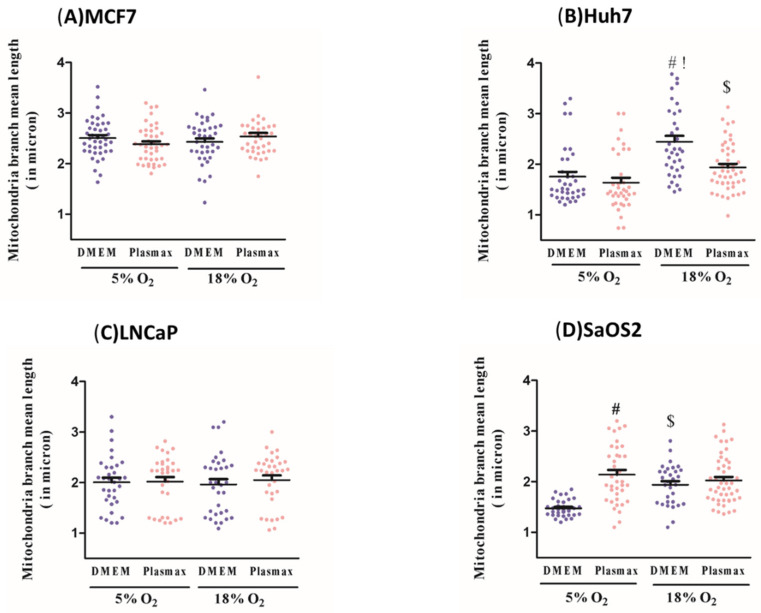
Oxygen concentration and media type influence mitochondrial branch size. Mitochondrial branch mean length (in microns) was measured using MiNA tool [16] in: (**A**) MCF7; (**B**) Huh7; (**C**) LNCaP; (**D**) SaOS2. Cells were cultured at either 5% O_2_ or 18% O_2_ in either DMEM or Plasmax for at least two weeks prior to commencing the measurements. Data shown are means ± SEM from 3 independent experiments per condition (*n* = 35 cells per each condition for each cell line). “#” represents differences between 18% O_2_ and 5% O_2_ in the same media. “$” represents differences between Plasmax and DMEM when oxygen level is the same. “!” represents differences between 5% O_2_/ Plasmax vs. 18% O_2_/DMEM. Statistical significance was determined by ANOVA followed by Tukey’s post-hoc analysis.

Neither media nor O_2_ level affected branch mean length (in microns), another proxy of mitochondrial fusion, in MCF7 or LNCaP cells (Figure 5A,C). However, at 5% O_2_ this value was greater in Plasmax than DMEM in SaOS2 cells (F_1, 136_ = 22, *p* <0.05, Figure 5D), and at 18% O_2,_ Huh7 cells in DMEM had an elevated branch mean length (F_1, 136_ = 23, *p* < 0.05, Figure 5B). Collectively, our data indicate that the media do not affect the mitochondrial branch mean length. Similarly, O_2_ effect on this measurement is not notable except for Huh7 and SaOS2 cell lines, having a larger branch mean length at 18% O_2_ when in DMEM (F_1, 136_ = 25; F_1, 136_ = 21, respectively; *p* < 0.05, Figure 5B,D). When comparing standard condition against the most physiologic one, Huh7 and SaOS2 cell lines had a higher branch mean length in the former (F_1, 136_ = 32; F_1, 136_ = 26, respectively; *p* < 0.05, Figure 5B,D) while in other cell types no significant difference was detected.

Collectively, we observed differences in energy metabolism (e.g., basal OCRs, ECAR) and mitochondrial morphology in response to media composition and O_2_ levels. These differences were mostly cell type-specific, which we attribute to the heterogeneity of cancer cells in general (reviewed in [20]).

## 4. Discussion

Our results clearly indicate the extent to which the energy metabolism of cancer cell lines is affected by culture conditions. In all four cell lines investigated, ECAR was significantly elevated when cells were growing in standard cell culture conditions, compared to physiologic media and O_2_ levels. This appeared to be driven by the culture media since, for any given O_2_ concentration, ECAR tended to be greater in DMEM than in Plasmax. Concomitant with this effect, in three of the four cell lines (MCF7, Huh7, LNCaP), basal and maximal OCR were lower in DMEM. Together, these results suggested that energy metabolism was shifted toward dependence on glucose fermentation to lactate in cells growing in DMEM, whereas cells growing in Plasmax had a more oxidative metabolism. Here, we used a formulation of DMEM containing 25 mM glucose, whereas Plasmax contains 5.5 mM glucose. Although most published papers do not report the actual formulation of DMEM used [5], and therefore not the concentration of glucose, it is likely that DMEM with 25 mM glucose is used commonly to avoid glucose depletion during multi-day cell culture. Although cancer cells are generally characterised by elevated rates of glucose consumption, this can be further promoted when they are grown in hyperglycemic media [21,22,23].

Increasing the reliance of cancer cells on glucose fermentation to lactic acid is problematic, particularly for experiments in which lactate dehydrogenase (LDH) is targeted. RNAi-mediated knockdown of the A-isoform of LDH, which favours pyruvate reduction to lactic acid, has toxic effects on several cancer cell lines (LDH knockdown inhibits in vitro and in vivo growth of various cancer cell lines [24,25,26]. Similar observations have been made in experiments targeting LDHA activity pharmacologically. For example, 1-(Phenylseleno)-4-(Trifuoromethyl) Benzene (PSTMB) inhibits LDHA activity while causing cell death in a range of cancer cell lines [27]. NCI-006 potently inhibits LDH in vitro and in vivo [28], and is considered a promising prospective drug for targeting tumour metabolism. Given the significant attention currently focused on metabolic inhibition strategies to combat cancers, it is particularly important to avoid non-physiological cancer cell culture approaches that alter energy metabolism in ways that may artifactually augment vulnerability to LDHA-inhibiting drugs.

It is important to note that, although the use of media modeled after the human blood plasma metabolome represents a step forward in the development of a more in vivo-like cell culture environment, the extracellular environment of many cancer cells in vivo is depleted of many nutrients relative to plasma (reviewed in [29]). For example, in murine pancreatic cancer tumours, interstitial fluid glucose concentration can be ~50% lower than in plasma [30]. Thus, arguably an even lower glucose concentration medium should be used in studies of LDH inhibitors, or indeed of any metabolic inhibitor.

It is surprising that, even in studies characterising physiologic media such as Plasmax and HPLM, experiments were performed at non-physiological O_2_. Hypoxia-inducible factor-1 (HIF-1) is a well-characterised mediator of aerobic glycolysis, via transcriptional stimulation of genes encoding glucose transporters and glycolytic enzymes (reviewed in [31]). Although the vast majority of studies examining the O_2_-regulated α-subunit (HIF-1α) have compared standard cell culture O_2_ (18%) with hypoxia (typically 1%), a more physiologically relevant comparison might be between 5% (physioxia) and 1% (hypoxia). Notably, HIF-1α is present in relatively low amounts in the physiological range from 2–5% O_2_ [32] where effects on transcription of glycolytic enzymes are likely significant. Alternatively, since cellular reactive oxygen species (ROS) production can be influenced by environmental O_2_ levels ([33]; reviewed in [34]) and concomitantly affects cellular metabolism and other functions, it is important to consider this parameter. Our data indicate that, within a given medium, O_2_ levels during cell culture can affect energy metabolism, apparent mitochondrial abundance and, to a lesser extent, mitochondrial network characteristics. Taken together, these effects of environmental O_2_ levels on metabolic and mitochondrial characteristics of cancer cells emphasise the importance of this variable in experimental design. As noted above for glucose, although we used 5% O_2_, which is typical of many normal human tissues [8], the O_2_ levels in tumours are often lower. Certainly, unregulated O_2_ that equilibrates to around 18% is inappropriate for cancer cell culture. Maintaining O_2_ at 5% or lower will better mimic the in vivo environment.

In summary, here we have described the metabolic effects of the cell culture environment on several well-studied cancer cell lines. These effects are substantial and may affect experiments aiming to identify or characterize metabolic inhibitors for targeting cancer cells in vivo. Given these observations, it is important to consider maintaining physiologically relevant nutrient concentrations, including O_2_, in vitro.

## Data Availability

Raw data are available on request from the corresponding authors.

## Abbreviations

DMEM	Dulbecco’s Modified Eagle Medium
ECAR	Extra cellular acidification
FBS	Fetal bovine serum
FCCP	Carbonyl cyanide-4-(trifluoromethoxy) phenylhydrazone
HPLM	Human Plasma-Like Medium
LDH	Lactate dehydrogenase
MiNA	Mitochondrial Network Analysis
OCR	Oxygen consumption rate
ROS	Reactive oxygen species
